# Function of Survivin in Trophoblastic Cells of the Placenta

**DOI:** 10.1371/journal.pone.0073337

**Published:** 2013-09-19

**Authors:** Cornelia Muschol-Steinmetz, Alexandra Friemel, Nina-Naomi Kreis, Joscha Reinhard, Juping Yuan, Frank Louwen

**Affiliations:** 1 Department of Gynecology and Obstetrics, School of Medicine, J. W. Goethe-University, Frankfurt, Germany; 2 Department of Gynecology and Obstetrics, St. Marien Hospital, Frankfurt, Germany; Medical Faculty, Otto-von-Guericke University Magdeburg, Medical Faculty, Germany

## Abstract

**Background:**

Preeclampsia is one of the leading causes of maternal and perinatal mortality and morbidity worldwide and its pathogenesis is not totally understood. As a member of the chromosomal passenger complex and an inhibitor of apoptosis, survivin is a well-characterized oncoprotein. Its roles in trophoblastic cells remain to be defined.

**Methods:**

The placental samples from 16 preeclampsia patients and 16 well-matched controls were included in this study. Real-time PCR, immunohistochemistry and Western blot analysis were carried out with placental tissues. Primary trophoblastic cells from term placentas were isolated for Western blot analysis. Cell proliferation, cell cycle analysis and immunofluorescence staining were performed in trophoblastic cell lines BeWo, JAR and HTR-8/SVneo.

**Results:**

The survivin gene is reduced but the protein amount is hardly changed in preeclamptic placentas, compared to control placentas. Upon stress, survivin in trophoblastic cells is phosphorylated on its residue serine 20 by protein kinase A and becomes stabilized, accompanied by increased heat shock protein 90. Depletion of survivin induces chromosome misalignment, abnormal centrosome integrity, and reduced localization and activity of Aurora B at the centromeres/kinetochores in trophoblastic metaphase cells.

**Conclusions:**

Our data indicate that survivin plays pivotal roles in cell survival and proliferation of trophoblastic cells. Further investigations are required to define the function of survivin in each cell type of the placenta in the context of proliferation, differentiation, apoptosis, angiogenesis, migration and invasion.

## Introduction

Survivin, a well-characterized oncoprotein, is best known for its participation in the chromosomal passenger complex (CPC), its capability to inhibit apoptosis and its involvement in the cellular stress response [1,2]. The gene expression of survivin is controlled by many cell signaling pathways at transcriptional and post-transcriptional levels [1,3,4]. While several oncogenic factors stimulate expression of the survivin gene, tumor suppressors repress it [5]. Survivin is located in the cytosol, mitochondria and nucleus [6,7], which is tightly linked to its various cellular functions. While the nuclear pool mediates its mitotic role, the cytosolic and mitochondrial fractions are responsible for its anti-apoptotic capability [7,8]. In response to apoptotic stimuli, survivin is trafficked from the mitochondria to the cytosol where it can inhibit apoptosis [7].

Survivin acts as an important regulatory member of the CPC in mitosis [9]. It is involved in proper chromosome alignment, spindle assembly, spindle stability via the suppression of microtubule dynamics [10] and kinetochore-microtubule attachment [11]. In mitosis, survivin is precisely regulated by Aurora B, Polo-like kinase 1 (Plk1) and cyclin-dependent kinase 1 (Cdk1) by phosphorylating its residues T117, S20 and T34, respectively [12–15]. Interfering with these regulations results in misaligned chromosomes, malattachment of the microtubule-kinetochore and defective cytokinesis [13–15]. In addition, survivin is highly expressed in various cancers and is linked to malignant progression, metastasis, therapy resistance and poor prognosis of patients [2].

Interestingly, survivin has been reported to be overexpressed in hydatidiform mole and choriocarcinoma [16,17]. Survivin promotes trophoblast survival by showing decreased cell viability and increased apoptosis in choriocarcinoma cell lines treated with antisense oligonucleotides [18]. While a higher level of survivin at the murine feto-maternal interface was suggested to be involved in pregnancy loss, upregulated survivin was proposed to support trophoblast survival and thus maintain pregnancy during placentation [19].

The expression level of survivin in preeclamptic placentas has also been controversially reported [20,21]. Preeclampsia, characterized by the new onset of hypertension and proteinuria after 20 weeks of gestation, is a complex disorder manifested by impaired implantation, endothelial dysfunction and systemic inflammation [22,23]. It affects 2–8% of all pregnancies and is one of the leading causes of maternal and perinatal mortality and morbidity worldwide [24]. Despite intensive research, its pathogenesis is not totally understood [22–25]. In our previous work, based on our own designed gene arrays (manuscript submitted), we observed that the gene coding for survivin was reduced in preeclamptic placenta compared to control. The aim of this study is to verify the data using quantitative real-time PCR and immunohistochemistry in bigger collectives, and to study the molecular function of survivin in trophoblastic cells of the placenta.

## Materials and Methods

### Sample collection

This study was approved by the Ethics Committees at Frankfurt University Hospital. Written informed consent was obtained from preeclampsia patients and controls. Preeclampsia was diagnosed as described [26]. Placenta samples (0.5 cm^3^) were taken from the four quadrants of the fetal side of placentas within 30 minutes post-delivery, frozen immediately in liquid nitrogen and stored at -80°C until use.

### RNA extraction, real-time PCR and data analysis

Total RNAs were extracted with RNeasy kits with column DNase digestion according to the manual instructions (QIAGEN, Hilden). Reverse transcription was performed using High-Capacity cDNA Reverse Transcription Kit as instructed (Applied Biosystems, Darmstadt). The primers and probes for survivin and Aurora B were obtained from Applied Biosystems. Real-time PCR was performed with a StepOnePlus Real-time PCR System (Applied Biosystems). The data were analyzed using StepOne Software v2.2.2 (Applied Biosystems). Using the comparative CT method [27], the gene expression was represented as ΔCt, which is normalized to endogenous controls and is reversely related to the amount of target molecules in the reaction. The mean value of expression levels of three genes SDHA (succinate dehydrogenase complex, subunit A), YWHAZ (tyrosine 3-monooxygenase/tryptophan 5-monooxygenase activation protein, zeta polypeptide) and TBP (TATA box-binding protein) served as endogenous controls [28]. The final results were represented as relative quantification (RQ), the difference in survivin expression level between preeclampsia samples and normal placenta samples, by setting the expression values of the normal placenta as 1, in medians with minimum and maximum range. The RQ of a group is defined as 2^-(ΔCTgroup-ΔCTcontrol)^, therefore, the RQ value for the control group itself leads to the value RQ=1 without variation. In the bar chart the RQ value of the control group is omitted and served just as a benchmark.

### Immunohistochemistry and Western blot analysis of placenta tissues

Formalin-fixed, paraffin-embedded placenta tissue sections were deparaffinized and further treated with 4% (v/v) hydrogen peroxide in TRIS-buffered saline (TBS) for 15 minutes. Antigen retrieval was carried out by exposure to pressure cooker conditions for 20 minutes with 10 mM citrate buffer, pH 6.0. Nonspecific binding was blocked with blocking buffer (Santa Cruz Biotechnology, Heidelberg) for 1 hour at room temperature. Sections were then incubated with rabbit monoclonal anti-survivin antibody (1:150, Cell Signaling, Beverley) overnight at 4°C and additional 30 minutes at room temperature. Non-immune rabbit IgG (Santa Cruz Biotechnology) was taken as negative control. After washing with TBS, sections were incubated with biotinylated goat anti-rabbit antibodies (Santa Cruz Biotechnology) for 40 minutes at room temperature, followed by washing with TBS. After incubation in a streptavidin-peroxidase conjugate, the antibody complexes were visualized through exposure to 3-amino-9-ethylcarbazole (AEC) substrate (DAKO, Hamburg) for 3 minutes. Sections were counterstained with hematoxylin, mounted and examined using an Axio Imager 7.1 microscope (Zeiss, Göttingen). Semi-quantitative analysis (HScore) of survivin staining was performed based on the combination of staining intensity with the percentage of positive cells [21]. Briefly, no staining was scored as 0, 1–10% of positive cells stained scored as 1, 10–50% as 2, 50–80% as 3, and 80–100% as 4. Staining intensity was rated on a scale of 0–3, with 0=negative, 1=weak, 2=moderate and 3=strong. The raw data were converted by multiplying the quantity and staining intensity scores. Negative controls were stained with non-immune antibody.

Cellular lysates from placental tissues were performed using total protein extraction kits from Millipore (Schwalbach), as instructed. For the extraction of cytoplasmic and nuclear proteins from placental tissues, a compartmental protein extraction kit was used (Millipore). Western blot analyses with tissue extracts were carried out as described [29,30]. Primary polyclonal rabbit antibody against survivin was from R&D systems (1:750, Minneapolis).

### Primary trophoblast isolation

Primary trophoblasts were isolated from term placentas as described [31]. Briefly, 50 g placenta tissue was finely minced, rinsed and digested with trypsin (Life Technology, Darmstadt) and DNase I (Sigma-Aldrich, Taufkirchen) for 20 min at 37°C on a shaker. The digestion was repeated two more times. The suspension was centrifuged at 1000 g for 10 min, resuspended and filtered with a 100 µm nylon cell strainer. The filtered suspension was again centrifuged before laying on two performed percoll density gradients ranging from 70% to 5%. The gradients were centrifuged and the cells ranging between the density 1.048 and 1.064 were collected. After centrifugation and counting, cells were seeded at a density of 2 x 10^5^ cells per cm^2^ and cultured overnight with IMDM medium (Life Technology). The purity of isolated primary trophoblasts from term placentas was analyzed by using both indirect immunofluorescence staining and immunocytochemistry with antibodies against vimentin (1:100, absent, for non-trophoblasts), cytokeratin-7 (1:100, positive for both cytotrophoblasts and syncytiotrophoblasts) and cytokeratin-18 (1:50, positive for cytotrophoblasts). Cells were then stimulated and harvested for Western blot analysis.

### Cell culture, cell cycle analysis, Western blot analysis and immunofluorescence staining

BeWo, JAR and HTR-8/SVneo (HTR) [32] cells were cultured as instructed. H_2_O_2_ and YM155 were obtained from Merck (Darmstadt) and forskolin from Sigma-Aldrich. Cell cycle profile was analyzed using a FACSCalibur (BD Biosciences, Heidelberg), as described [33]. Briefly, cells were harvested, washed with PBS, fixed in chilled 70% ethanol at 4°C for at least 30 min, treated with 1 mg/ml of RNase A (Sigma-Aldrich) and stained with 100 µg/ml of propidium iodide for 30 min at 37°C. DNA content was determined by FACS. The data were analyzed with CellQuest software (BD Biosciences).

Cell lysis was performed using RIPA buffer (50 mM Tris-HCl pH 8.0, 150 mM NaCl, 1% NP-40, 0.5% Na-desoxycholate, 0.1% SDS, 1 mM NaF, 1 mM DTT, 0.4 mM PMSF, 0.1 mM Na _3_VO_4_, and protease inhibitor cocktail complete (Roche, Mannheim)). Western blot analysis was performed, as previously described [34]. The following antibodies were used for Western blot analysis: mouse monoclonal antibodies against Plk1, p53 and Bax (1:1000, 1:500 and 1:500, respectively, Santa Cruz Biotechnology), rabbit polyclonal antibody against phospho-p53 (S15) (1:500, Cell Signaling), rabbit polyclonal antibody against survivin (1:750, R&D Systems), rabbit polyclonal antibodies against p21 and against poly(ADP-ribose) polymerase (PARP) (both 1:1000, Cell Signaling), rabbit polyclonal antibody against phospho-survivin (S20) (1:1000, Novus Biologicals, Cambridge), mouse monoclonal antibody against heat shock protein 90 (Hsp90) (1:1000, Abcam, Berlin), mouse monoclonal antibody against Bcl-2 (1:500, Millipore) and mouse monoclonal antibodies against β-actin (1:200,000, Sigma-Aldrich, Taufkirchen) and lamin B1 (1:1000, MDL, Woburn).

Indirect immunofluorescence staining was performed as described [29,30,34]. In brief, control or treated cells were fixed for 15 min with 4% PFA containing 0.1% Triton ^®^X-100 at room temperature. The following primary antibodies were used for staining: polyclonal rabbit antibody against pericentrin (1:800, Abcam), immune serum against centromere (1:400, anti-centromere antibody, ACA, ImmunoVision), polyclonal rabbit antibody against survivin (1:1000, R&D Systems), polyclonal rabbit antibody against Aurora B (1:150, Cell Signaling), monoclonal mouse antibodies against vimentin and cytokeratin-7 (both 1:100, DAKO), monoclonal rabbit antibody against cytokeratin-18 (1:50, Abcam) and FITC-conjugated mouse monoclonal antibody against α-tubulin (1:500, Sigma-Aldrich). DNA was stained using DAPI (4’,6-diamidino-2-phenylindole-dihydrochlorid) (Roche). Slides were examined using an Axio Imager 7.1 microscope (Zeiss) and images were taken using an Axio Cam MRm camera (Zeiss). The immunofluorescence stained slides were also examined by a confocal laser scanning microscope (CLSM) (Leica CTR 6500, Heidelberg). Images were processed using Photoshop.

### siRNA transfection, active caspase-3/7 measurement and cell proliferation assay

siRNA targeting survivin (sense: GCAGGUUCCUUAUCUGUCA and antisense UGACAGAUAAGGAACCUGC) was manufactured by Sigma-Aldrich. Control siRNA was obtained from Qiagen. siRNA (20 nM, unless otherwise stated) was transiently transfected using transfection reagent Oligofectamine^TM^ as instructed (Life Technology). The activity of caspase-3/7 was analyzed with Caspase-Glo^®^ 3/7 Assay (Promega GmbH, Mannheim). Cell proliferation assays were carried out by using Cell Titer-Blue^®^ Cell Viability Assay on treated cells in 96-well plates, based on the reduction of the indicator dye Resazurin into Resorufin by viable cells (Promega GmbH). 20 µl of CellTiter-Blue^®^ reagent was added to each well and then incubated at 37°C with 5% CO_2_ for 3 h before fluorescence reading using a Victor 1420 Multilabel Counter (Wallac). Experiments were performed in triplicate. Data were presented as percentages compared to control.

### Statistical analysis

The student’s t-test (two tailed and paired) was used to evaluate the significance of difference between two groups with normal distribution. Otherwise the non-parametric Mann-Whitney U-Test was used between the patient group and control group. Difference was considered as statistically significant when p< 0.05.

## Results

To select matched normal controls for preeclampsia patients, special attention was paid to gestational age, delivery mode, body mass index (BMI) and maternal age. The clinical data of 16 preeclampsia patients and 16 well-matched controls are summarized in Table 1. There was no significant difference in gestational age, maternal age and BMI between normal pregnancies and those complicated by preeclampsia.

**Table 1:** Clinical information of 16 preeclampsia patients and 16 controls.

	age	gestational age (weeks)	body mass index	birth weight (g)	systolic BP (mmHg)	diastolic BP (mmHg)	proteinuria (mg/24h)
control	33.6±6.4	37.0±4.2	26.6±3.8	3022±899	126±16	74±13	0
preeclampsia	31.6±4.2	36.8±3.9	26.4±4.9	2650±742	167±27	101±14	1792±2185
P value	0.28	0.93	0.86	0.16	0.00001	0.00001	-

### The mRNA level of survivin was decreased in preeclamptic placentas

**Figure 1:**
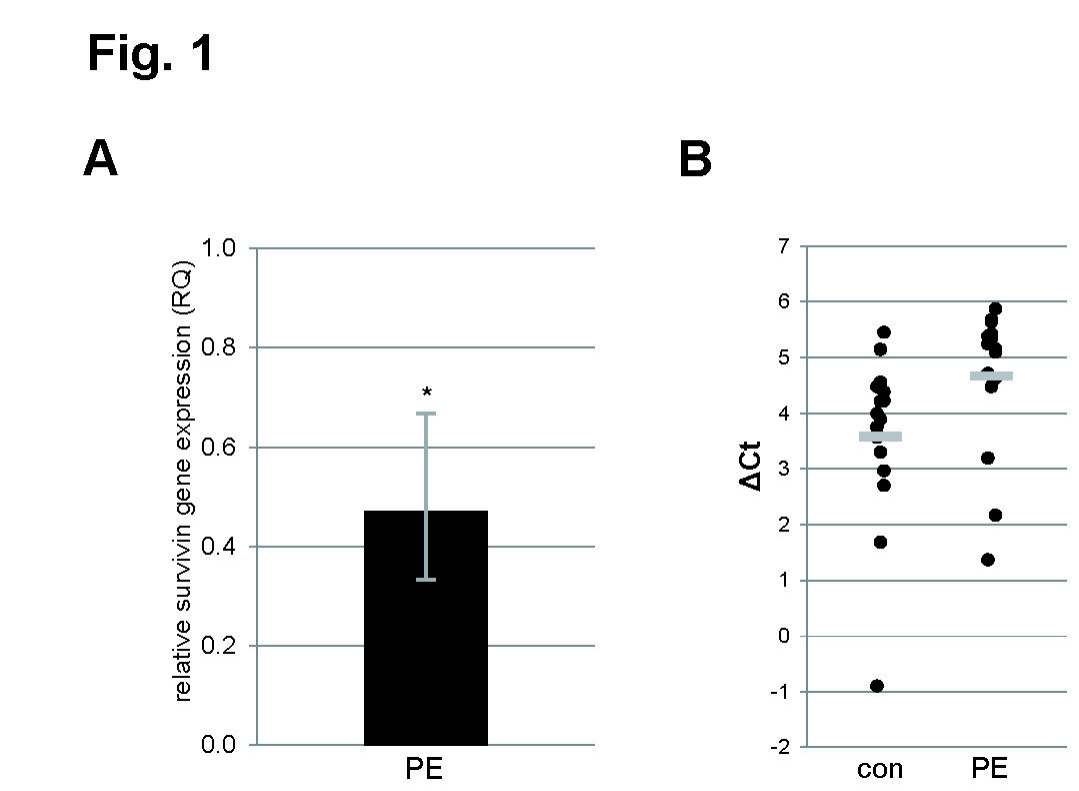
Survivin gene is reduced in preeclamptic placentas. (**A**) Relative amount of the gene survivin in preeclamptic placentas (PE, n=16), in comparison with control placentas (n=16). The results are presented by mean of RQ with minimum and maximum range and statistically analyzed between control and PE group. *p < 0.05. RQ: relative quantification of fold by setting the control value as 1, as described in the Materials and Methods section. The RQ value of the control group is omitted and served just as a benchmark. (**B**) Individual expression levels of the survivin gene in both preeclampsia and control groups, represented by ΔCt values, which are normalized to endogenous controls and are reversely related to the amount of target molecules, as described in the Materials and Methods section. Bar: median value.

According to the instruction of the comparative CT analysis protocol [27], the expression values of the normal control placentas was set as 1. The mRNA level of survivin in preeclamptic placentas was significantly decreased, only 45.2% of that in normal placentas (Figure 1A), which is consistent with our previous data (manuscript submitted). The relative mRNA levels of survivin of both groups are illustrated in Figure 1B.

### The protein level of survivin was hardly changed in preeclamptic placentas

**Figure 2:**
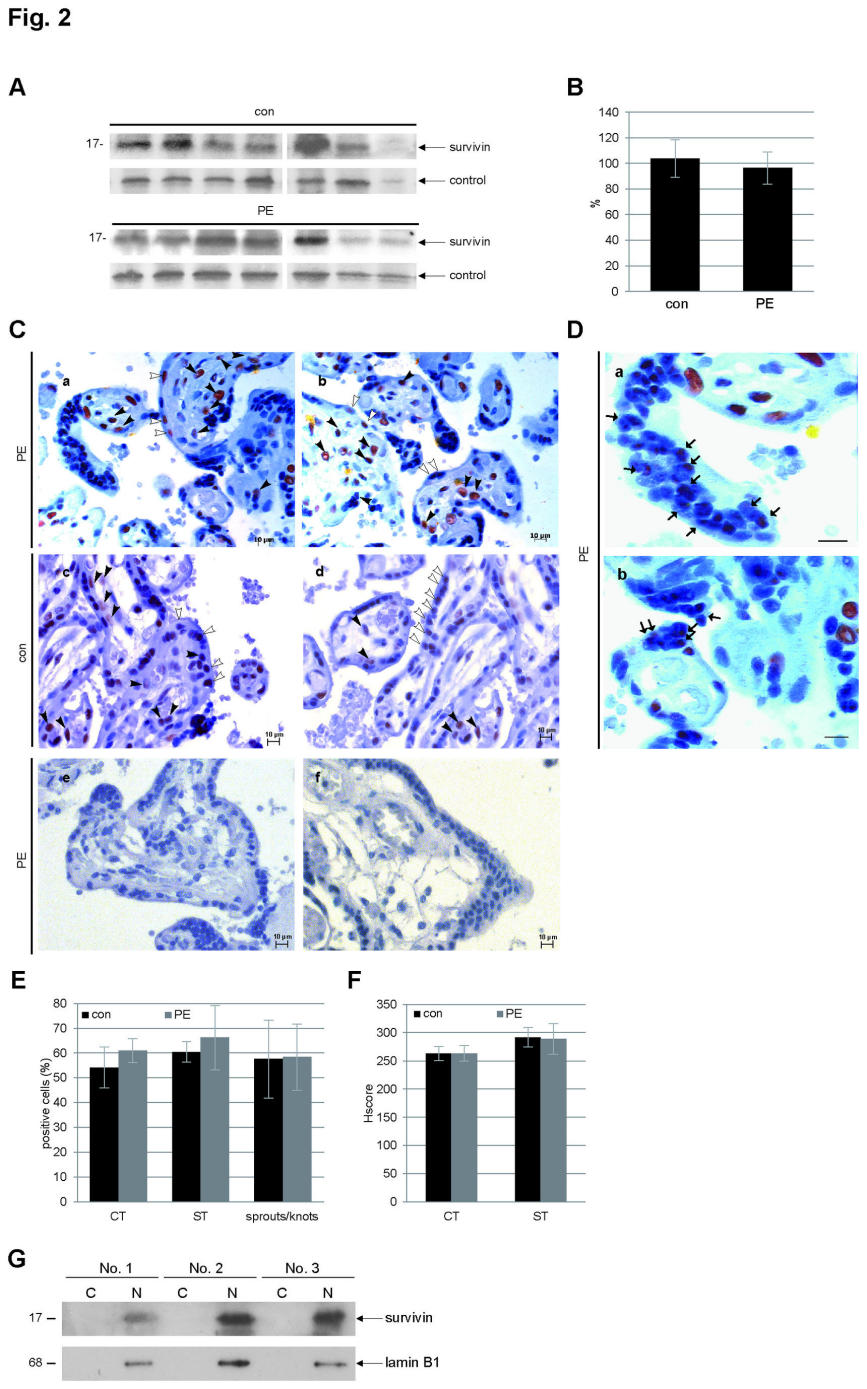
Survivin protein is comparable between preeclampsia and control group and is located in the nucleus. (**A**) Western blot analysis of control placentas (con, n=16) and preeclamptic placentas (PE, n=16). The representatives are shown. (**B**) Quantification of the relative amounts of survivin protein in the control group and in the preeclampsia group from Western blot analysis. Bar: standard deviation (± SD). (**C**) The representatives of immunohistochemical staining with survivin antibody in preeclampsia placentas (a and b) and in control placentas (c and d). Open arrowheads and closed arrowheads indicate some of stained syncytiotrophoblasts and cytotrophoblasts, respectively. Immunohistochemical stainings with control IgG or survivin antibody neutralized with corresponding peptide are presented as controls (e and f). Scale bar: 10 µm. (**D**) Clear signals of survivin in syncytial sprout cells (black arrows in a, magnification of Figure 2C, a) and signals in syncytial knots (black arrows in b). Scale bar: 10 µm. (**E**) Quantification of positive cells of survivin staining in 7 placenta samples from PE patients and 7 samples from matched controls (n=1500 cells per cell type). CT: villous cytotrophoblasts. ST: syncytiotrophoblasts. Sprouts/knots: syncytial sprout cells and syncytial knots. Bar: ± SD. (**F**) Quantification of survivin positive rate using HScore method, as described in the Materials and Methods section, in villous cytotrophoblasts (CT) and syncytiotrophoblasts (ST). Bar: ± SD. con: control placenta. PE: preeclamptic placenta. (**G**) Western blot analysis using cytosol (C) or nuclear extracts (N) from placentas. Lamin B1 served as nuclear extract marker.

Unexpectedly, the protein levels of survivin between the two groups were not obviously different (Figure 2A). Further quantification of the survivin bands in Western blots showed no significant alteration in the protein level between the preeclampsia group and the control group (Figure 2B). By immunohistochemical staining, survivin was to be clearly found in villous cytotrophoblasts and syncytiotrophoblasts (Figure 2C, a-d). The signal was not observed in placenta tissue stained with either control IgG (Figure 2C, e) or survivin antibody neutralized with corresponding peptide (Figure 2C, f), indicating that the staining signal of survivin is specific. Interestingly, the survivin signal was predominant in the nucleus but also to be found in the cytoplasm (Figure 2C, a-d). Of note, the survivin staining was easily detectable in the nuclei of syncytial sprout cells (Figure 2D, a). In addition, the staining signal was also observable in syncytial knot cells (Figure 2D, b) in both preeclamptic placentas as well as in normal placentas. Further evaluation in each subtype of placental cells showed that the percentages of positive staining of survivin in villous cytotrophoblasts and syncytiotrophoblasts were even slightly, although not significantly, higher in preeclamptic placentas than in matched control placentas (Figure 2E). Finally, according to HScore evaluation, described in the Materials and Methods section, there was no difference of survivin staining in villous cytotrophoblasts and syncytiotrophoblasts between preeclampsia placentas and control placentas (Figure 2F). To corroborate the predominant nuclear localization of survivin observed in immunohistochemical staining, we separated cytosol and nuclear extracts of placenta tissues and performed Western blot analysis using another specific antibody against survivin, not the antibody used in immunohistochemistry. As shown in Figure 2G, it was obvious that the major pool of survivin was found in nuclear extracts of placental tissues.

### Survivin was stabilized and its residue serine 20 is phosphorylated upon stress

**Figure 3:**
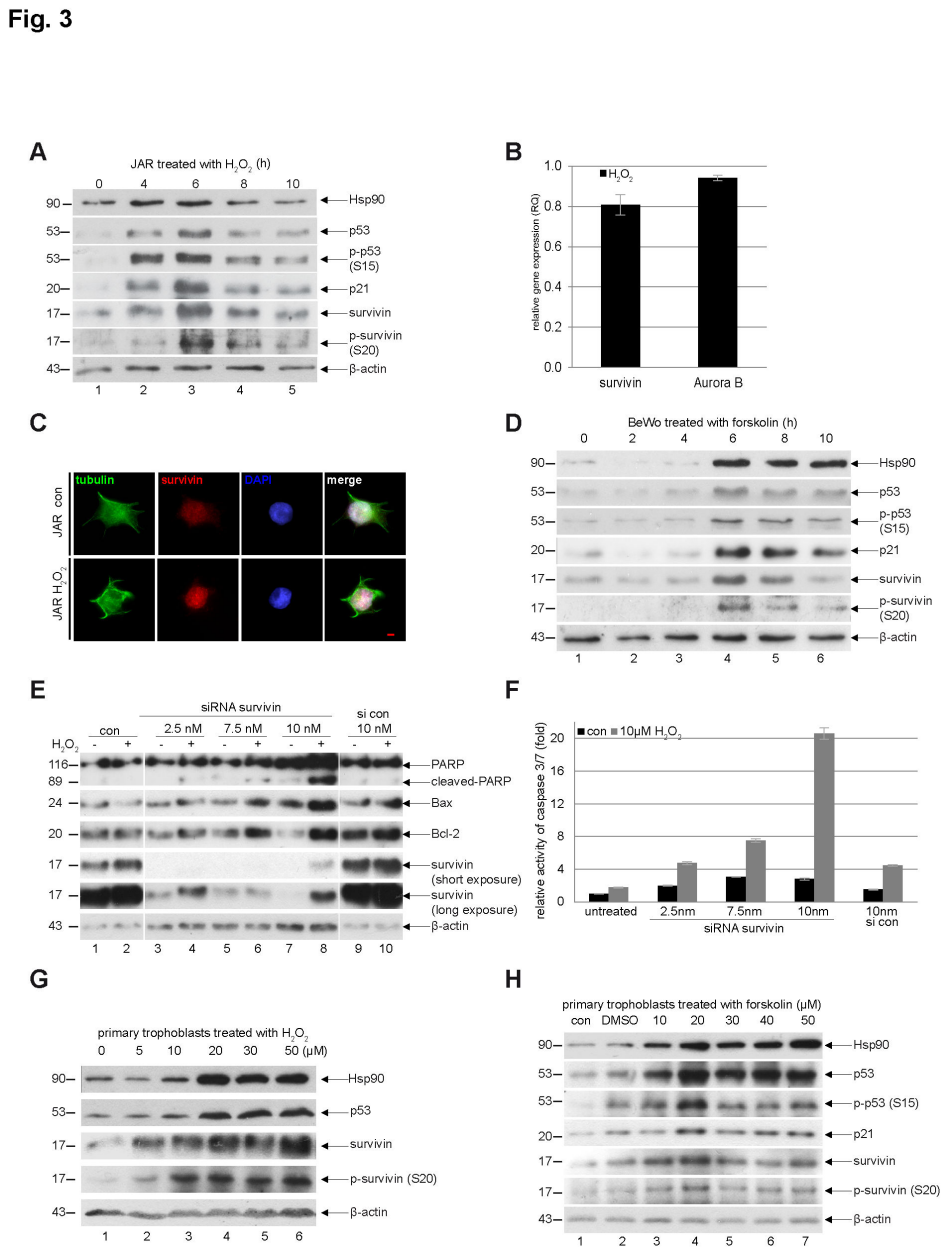
Survivin is stabilized and protects trophoblastic cells from apoptosis upon stress. (**A**) Western blot analysis. JAR cells were treated with 10 µM H_2_O_2_ and cellular lysates were prepared at 0, 4, 6, 8 and 10 h for Western blot analysis using antibodies as indicated. β-actin served as loading control. (**B**) Gene levels of survivin and Aurora B. Cells were treated as in (A) for 6 h and total RNA was isolated for real-time PCR analysis. The results are based on three independent experiments and presented as mean ± SEM. The RQ value of the untreated control cells is omitted and served just as a benchmark. (**C**) Immunofluorescence staining. JAR cells were treated with H_2_O_2_ for 6 h, fixed and stained for tubulin, survivin and DNA. Scale bar: 5 µm. (**D**) BeWo cells were treated with 20 µM forskolin for 0, 2, 4, 6, 8 and 10 h and cellular extracts were prepared for Western blot analysis with indicated antibodies. β-actin served as loading control. (**E**) Western blot analysis. JAR cells were non-treated (con), treated with scrambled siRNA (si con) or with siRNA targeting survivin (2.5, 7.5 and 10 nM) for 18 h and were then challenged with 10 µM H_2_O_2_ for additional 12 h. Cellular lysates were prepared for Western blot analysis with indicated antibodies. β-actin served as loading control. (**F**) Relative activity of caspase-3/7. The lysates from JAR cells treated as in (F) were used for evaluating caspase-3/7 activity. The results are presented as mean ± SD. (**G**) Western blot analysis. Primary trophoblasts were isolated from term placentas and stimulated with indicated concentrations of H_2_O_2_ for 6 h and cellular lysates were prepared for Western blot analyses with antibodies as indicated. β-actin served as loading control. (**H**) Western blot analysis. Isolated term primary trophoblasts were treated with increasing concentrations of forskolin for 8 h and cellular extracts were prepared for Western blot analyses with indicated antibodies. β-actin served as loading control.

We were wondering why the discrepancy exists between the gene expression and protein level of survivin in preeclamptic placentas. It is well known that transcript abundances only partially predict protein abundances because of substantial regulatory processes occurring after mRNA is translated [35–37]. Preeclamptic placenta is characterized by ischemia and hypoxia and it releases variety of stress mediators including reactive oxygen species (ROS), such as hydrogen peroxide (H_2_O_2_) [38]. Trophoblastic cells in the preeclamptic placentas encounter therefore perpetually enormous cellular stress and we asked whether the stress could modify the protein survivin and affect its turnover. To clarify this issue, we analyzed the protein stability of survivin upon stress in trophoblastic cells. JAR cells, trophoblastic cells derived from choriocarcinoma, were stimulated with H_2_O_2_ for indicated time periods and cellular lysates were prepared for Western blot analysis. As displayed in Figure 3A, H_2_O_2_ apparently stabilized survivin at 4 h and reached its peak at 6 h (Figure 3A, 5^th^ row), accompanied by increased p53 (Figure 3A, 2^nd^ row), phospho-p53 (Figure 3A, 3^rd^ row) and p21 (Figure 3A, 4^th^ row), the stress indicators. In particular, the residue serine 20 (S20) in survivin was phosphorylated (Figure 3A, 6^th^ row) and heat shock protein 90 (Hsp90) was elevated upon H_2_O_2_ treatment (Figure 3A, 1^st^ row). The mRNA levels of survivin and Aurora B at 6 h were not significantly elevated (Figure 3B). To look at subcellular localization of stabilized survivin, the treated cells were fixed and stained for tubulin, survivin and DNA. The localization of survivin was not altered but intensity of the signal was slightly increased after H_2_O_2_ treatment (Figure 3C).

It has been reported that the residue S20 in survivin is phosphorylated by both protein kinase A (PKA) [39] and Polo-like kinase 1 (Plk1) [14]. As Plk1 is mainly active in mitosis, we assumed that phosphorylation of S20 in survivin could be ascribed to an activation of PKA in trophoblastic cells subjected to H_2_O_2_. To test this, BeWo cells, another trophoblastic cell line derived from choriocarcinoma, were treated with 20 µM forskolin, an activator of PKA. Treated cells were harvested at indicated time points for Western blot analysis. The residue S20 of survivin was indeed phosphorylated after forskolin treatment (Figure 3D, 6^th^ row), associated with stabilized survivin (Figure 3D, 5^th^ row) and elevated Hsp90 (Figure 3D, 1^st^ row). In addition, the stress indicators were also increased, including p53, p-p53 and p21 (Figure 3D, 2^nd^ to 4^th^ rows). The same experiment was also carried out in HTR-8/SVneo cells, immortalized normal 1^st^ term trophoblast cells, and similar results were observed (data not shown).

To investigate the survival role of survivin in trophoblastic cells, JAR cells were treated with siRNA targeting survivin (2.5, 7.5 and 10 nM) for 18 h. Cells were then challenged by the stress inducer H_2_O_2_ for 12 h and cellular extracts were prepared for Western blot analysis. Survivin was efficiently depleted in JAR cells in a siRNA dose dependent manner (Figure 3E, 4^th^ row, lanes 3, 5 and 7). Again, H_2_O_2_ treatment stabilized the survivin protein (Figure 3E, 4^th^ row, lanes 2 and 10, 5^th^ row, lanes 4 and 8). Along with reduced survivin, cleaved poly(ADP-ribose) polymerase (PARP), an apoptosis marker, was enhanced (Figure 3E, 1^st^ row, lanes 4, 6 and 8) with increased pro-apoptotic protein Bax (Figure 3E, 2^nd^ row, lanes 4, 6 and 8). Intriguingly, the anti-apoptotic protein Bcl-2 was also enhanced (Figure 3E, 3^rd^ row, lanes 4, 6 and 8), which was not able to block the apoptotic response induced by H_2_O_2_. In consistence with these results, the caspase-3/7 activity in JAR cells treated with H_2_O_2_ was elevated, dependent inversely on remaining survivin amounts (Figure 3F).

Moreover, to corroborate these data, primary trophoblastic cells were isolated from term placentas. The characterization was carried out by staining the isolated primary cells with antibodies against vimentin, cytokeratin-7 and cytokeratin-18 (Figure S1). Primary trophoblasts were stimulated with H_2_O_2_ (5, 10, 20, 30 and 50 µM) for 6 h or with forskolin (10, 20, 30, 40 and 50 µM) for 8 h and cellular lysates were prepared for Western blot analysis. In accordance with the results from trophoblastic cell lines, survivin was clearly stabilized with H_2_O_2_ treatment (Figure 3G) and with forskolin (Figure 3H) in primary trophoblast cells, accompanied by increased levels of Hsp90 and p53. Collectively, the data indicate that, upon stress, survivin in trophoblastic cells is stabilized, which is associated with increased Hsp90 and activated PKA.

### Depletion of survivin inhibits proliferation of trophoblastic cells by arresting them in G2/M and by inducing apoptosis

**Figure 4:**
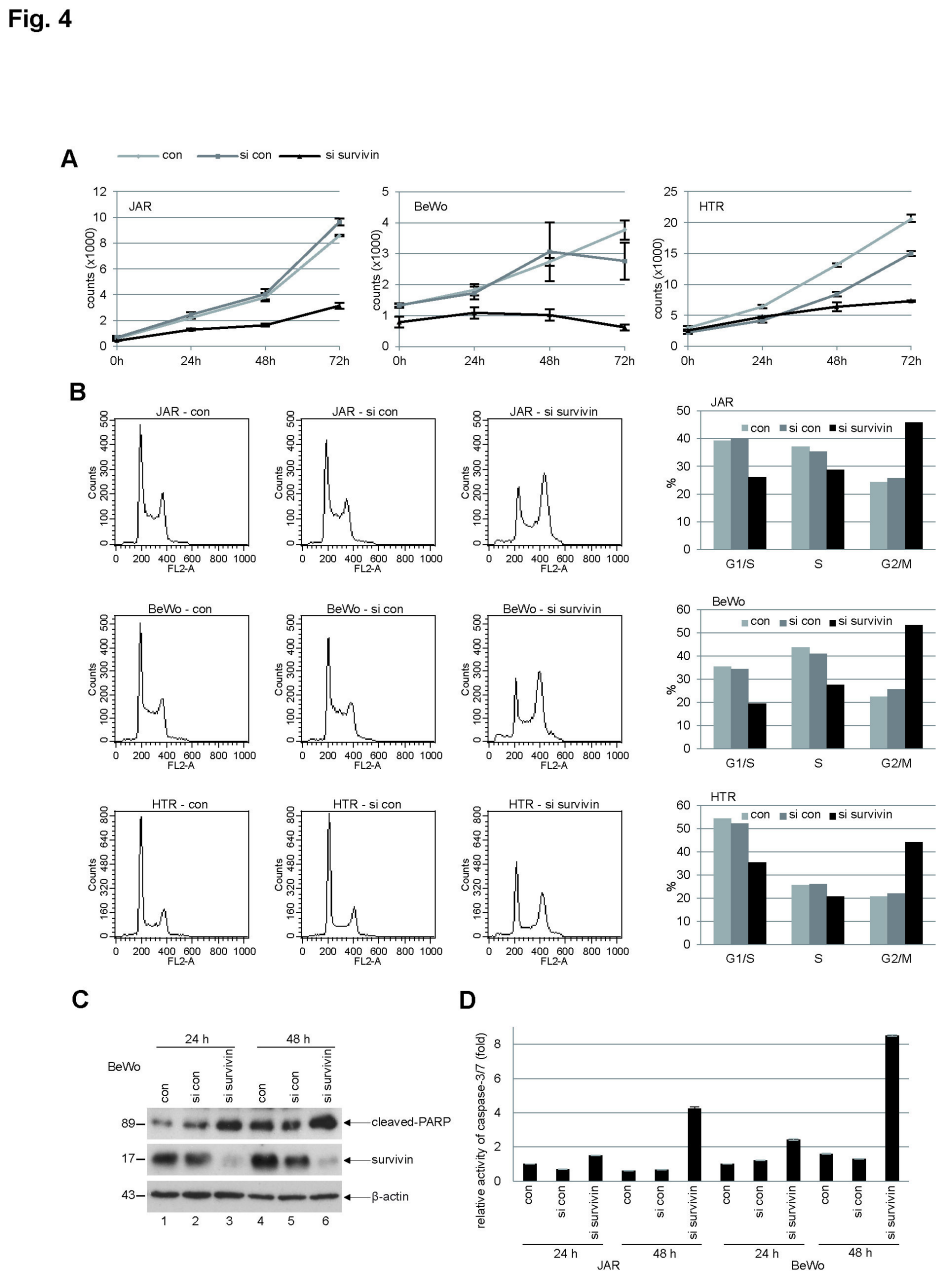
Survivin is required for the proliferation of trophoblastic cells and depletion induces a G2/M arrest. (**A**) Cell viability assay. JAR (left panel), BeWo (middle panel) and HTR-8/SVneo (HTR, right panel) cells were non-treated (con), treated with either control scrambled siRNA (si con) or siRNA targeting survivin (si survivin) and cell viability was analyzed at indicated time points. Bar: ± SD. (**B**) Cell cycle profiles. Cells were depleted of survivin for 48 h and cell cycle analysis was performed. con: untreated cells as control. si con: treated with control scrambled siRNA. si survivin: treated with siRNA against survivin. Right panels: quantification of the sub-phases of the cell cycle. (**C**) Western blot analysis. BeWo cells were transfected with scrambled siRNA (si con) or siRNA targeting survivin (si survivin) for 24 h or 48 h and cellular extracts were prepared for Western blot analysis using indicated antibodies. β-actin served as loading control. con: non-treated lysates as control. (**D**) Relative caspase-3/7 activity. The lysates from JAR or BeWo cells treated as in (C) were used for evaluating caspase-3/7 activity. The results are presented as mean ± SD.

As a member of the CPC, survivin promotes mitosis and therefore proliferation. To address the proliferative role of survivin in trophoblasts of the placenta, JAR, BeWo and HTR-8/SVneo were treated with siRNA targeting survivin and cell viability was then monitored. Regardless of whether normal or malignant, depletion of survivin disrupted proliferation of all three trophoblastic cell lines (Figure 4A), suggesting the importance of survivin for trophoblast division. Cell cycle analysis showed that trophoblast cells were arrested in G2/M upon deletion of survivin (Figure 4B). To explore if apoptosis took place after G2/M arrest, BeWo cells were treated with siRNA against survivin and cellular extracts were prepared for Western blot analysis. Cleaved PARP was increased in BeWo cells after treatment with siRNA against survivin (Figure 4C, 1^st^ row). In line with these results, the caspase-3/7 activity was strongly enhanced in BeWo and JAR cells treated with siRNA targeting survivin, particularly at 48 h (Figure 4D).

**Figure 5:**
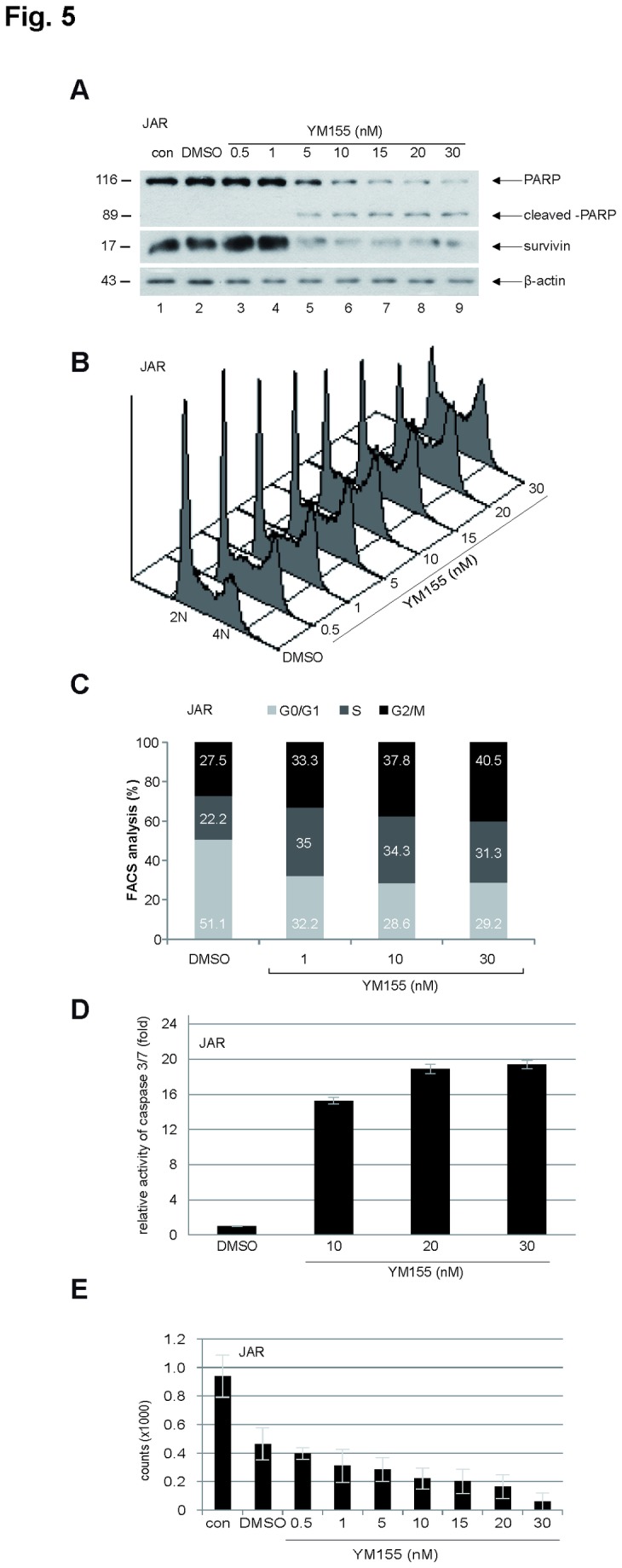
Trophoblastic cells are affected by survivin suppressant YM155. (**A**) Western blot analysis. JAR cells were treated with increasing concentrations of YM155 for 24 h and cellular extracts were prepared for Western blot analysis. β-actin served as loading control. Untreated (con) and DMSO treated cellular extracts were taken as controls. (**B**) Cell cycle profiles. Cells were treated as in (A) and cell cycle analysis was performed. DMSO treated cells were taken as controls. (**C**) Quantification of the sub-phases of the cell cycle. (**D**) Relative caspase-3/7 activity. The lysates from JAR cells treated with DMSO or 10, 20 and 30 nM YM155 for 24 h were used for evaluating caspase-3/7 activity. The results are presented as mean ± SD. (**E**) Cell viability assay. JAR cells were non-treated (con), treated with DMSO or with increasing concentrations of YM155 for 24 h and cell viability was analyzed. Bar: ± SD.

To verify the data, trophoblastic cells were incubated with YM155, a specific small molecule suppressant of the survivin expression [40]. The survivin level in treated JAR cells was convincingly reduced (Figure 5A), along with an increased peak of G2/M arrest (Figure 5B and C), a strongly enhanced induction of apoptosis (Figure 5A and D) and inhibited cell proliferation (Figure 5E). The same experiments were also carried out with BeWo cells and the similar results were obtained (Figure S2).

### Suppression of survivin resulted in misaligned chromosomes and abnormal centrosomes

**Figure 6:**
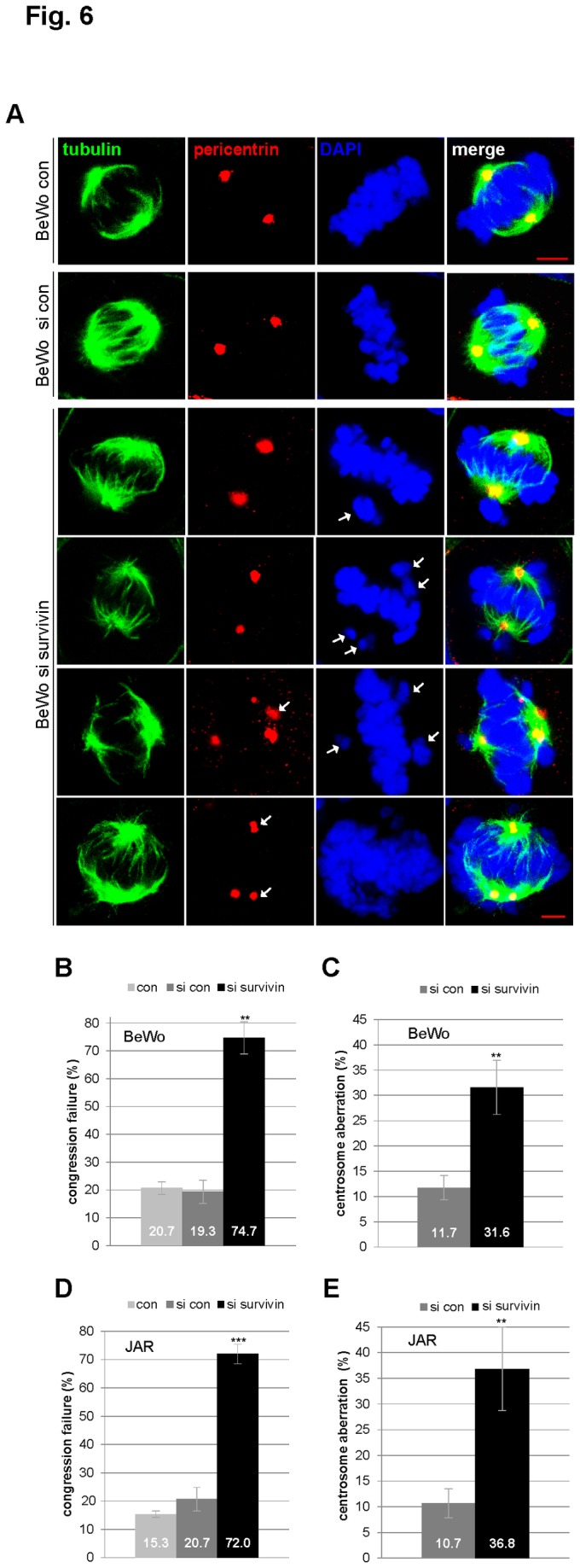
Survivin is required for chromosome alignment and centrosome integrity in trophoblastic cells. (**A**) BeWo cells were untreated (1^st^ panel), treated with scrambled siRNA (si con, 2^nd^ panel) or siRNA against survivin (si survivin, 3^rd^ to 6^th^ panels) for 36 h. Cells were then fixed and stained for tubulin, pericentrin and DNA, and analyzed by confocal laser scanning microscopy. Scale bar: 5 µm. Arrow: indicating either chromosome congression defect (DAPI staining) or failure of centrosome integrity (pericentrin staining). (**B**) Quantification of defects in chromosome congression in metaphase cells treated as in (A) (n=150 metaphase cells per condition). The results are presented as mean ± SD and statistically analyzed between siRNA control and siRNA survivin group. **p < 0.01. (**C**) Quantification of abnormal centrosomes in metaphase cells treated as in (A) (n=150 metaphase cells per condition). The results are presented as mean ± SD and statistically analyzed between siRNA control and siRNA survivin group. **p < 0.01. (**D**) The same experiments were also performed with JAR cells. Quantification of defect in chromosome congression in JAR metaphase cells treated as in (A) (n=150 metaphase cells per condition). The results are presented as mean ± SD and statistically analyzed between siRNA control and siRNA survivin group. ***p < 0.001. (**E**) Quantification of abnormal centrosomes in JAR metaphase cells treated as in (A) (n=150 metaphase cells per condition). The results are presented as mean ± SD and statistically analyzed between siRNA control and siRNA survivin group. **p < 0.01.

As an important regulatory member of the CPC, survivin plays multiple roles throughout mitosis. We were interested in the mitotic phenotype of trophoblastic cells in the absence of survivin. BeWo and JAR cells, depleted of survivin, were fixed and stained for microtubule marker tubulin, centrosome marker pericentrin and DNA. Metaphase cells were examined by confocal laser scanning microscopy. Compared to control cells (Figure 6A, 1^st^ and 2^nd^ panels), BeWo cells without survivin showed defects in chromosome congression (Figure 6A, 3^rd^ to 6^th^ panels, DAPI staining). Moreover, centrosome abnormality, more than two centrosomes yet often clustering into two poles, was observed (Figure 6A, 5^th^ and 6^th^ panels, pericentrin staining), often associated with abnormal spindle form (Figure 6A, 5^th^ panel, tubulin staining). Further evaluation showed that 74.7% of BeWo cells displayed misaligned chromosomes in the absence of survivin, compared to about 20% in control cells (Figure 6B). In addition, while 11.7% of control cells showed abnormal centrosomes, 31.6% of BeWo cells exhibited this defect after treatment with siRNA targeting survivin (Figure 6C). The same experiment was also performed in JAR cells and similar results were observed (Figure 6D and E).

### Reduced localization and activity of Aurora B in trophoblastic metaphase cells

**Figure 7:**
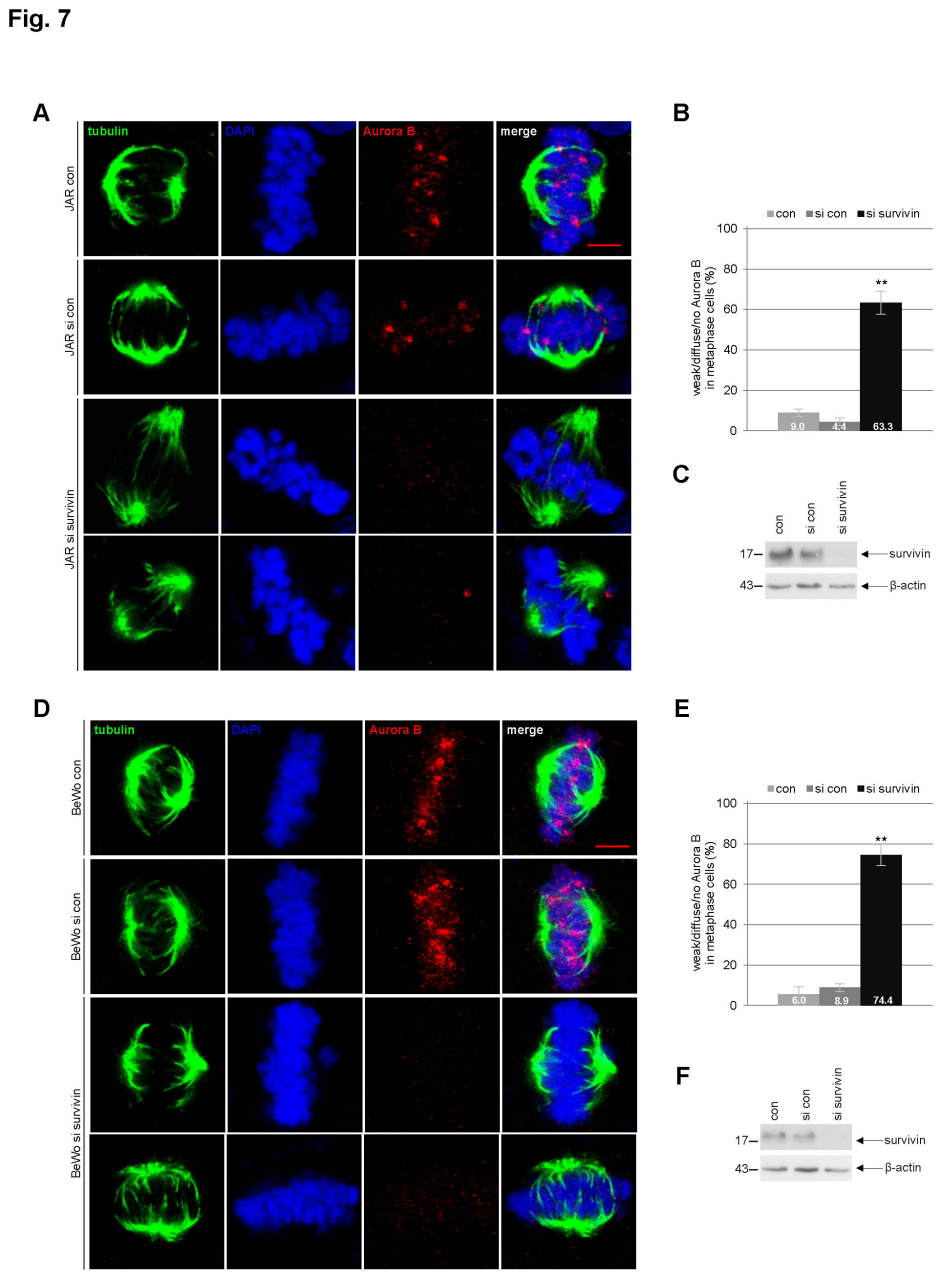
Localization of Aurora B at the centromeres/kinetochores is reduced after depletion of survivin. (**A**) JAR cells were untreated (1^st^ panel), treated with scrambled siRNA (si con, 2^nd^ panel) or siRNA against survivin (si survivin, 3^rd^ and 4^th^ panels) for 36 h. Cells were then stained for tubulin, DNA and Aurora B, and analyzed by confocal laser scanning microscopy. Scale bar: 5 µm. (**B**) Quantification of weak/no staining of Aurora B in metaphase cells treated as in (A) (n=90 metaphase cells per condition). The results are presented as mean ± SD and statistically analyzed between control and siRNA survivin group. **p < 0.01. (**C**) Western blot analysis as siRNA efficiency control. β-actin served as loading control. (**D**) BeWo cells were untreated (con, 1^st^ panel), treated with scrambled siRNA (si con, 2^nd^ panel) or siRNA against survivin (si survivin, 3^rd^ and 4^th^ panels) for 36 h. Cells were then stained for tubulin, Aurora B and DNA, and analyzed by confocal laser scanning microscopy. Scale bar: 5 µm. (**E**) Quantification of weak/no staining of Aurora B in BeWo metaphase cells treated as in (D) (n=90 metaphase cells per condition). The results are presented as mean ± SD and statistically analyzed between control and siRNA survivin group. **p < 0.01. (**F**) Western blot analysis as siRNA efficiency control. β-actin served as loading control.

As survivin is responsible for the localization of Aurora B at the centromere of the chromosome by binding directly to phospho-histone H3 (Thr-3) [41,42], we were next interested in the localization of Aurora B in mitotic trophoblast cells. JAR cells were depleted of survivin (Figure 7C) and stained for tubulin, DNA and Aurora B. Metaphase cells were evaluated by confocal laser scanning microscopy. Compared to control cells (Figure 7A, 1^st^ and 2^nd^ panels), JAR cells depleted of survivin displayed much weaker or no clear staining of Aurora B at the centromeres/kinetochores in metaphase (Figure 7A, 3^rd^ and 4^th^ panels). The localization of Aurora B was deregulated in over 60% of JAR cells in the absence of survivin (Figure 7B). The same experiment was also carried out in BeWo cells (Figure 7D and F) and over 70% of BeWo cells deficient in survivin demonstrated a defect in Aurora B localization at the centromeres/kinetochores (Figure 7E).

**Figure 8:**
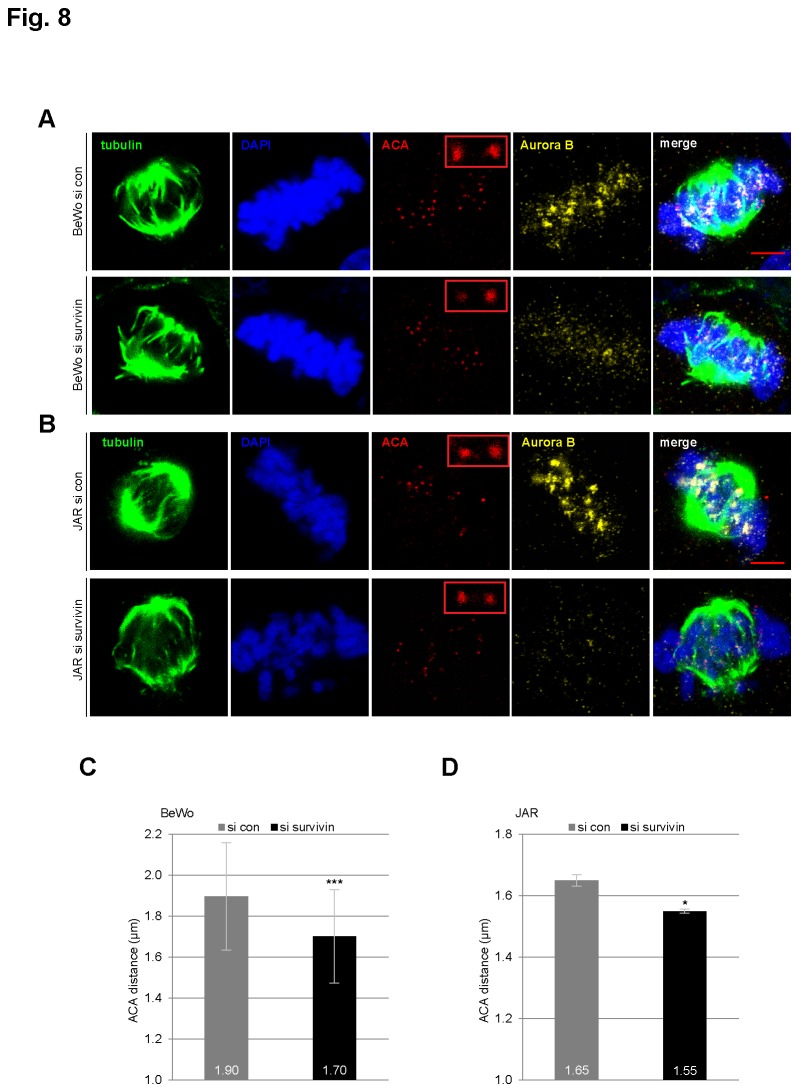
The distance of the sister centromeres is reduced in trophoblastic cells depleted of survivin. (**A**) BeWo cells were treated with scrambled siRNA (si con, upper panel) or siRNA against survivin (si survivin, lower panel) for 36 h. Cells were then stained for tubulin, DNA, the centromere with anti-centromere antibody (ACA) and Aurora B. The stained metaphase cells were analyzed by confocal laser scanning microscopy. Scale bar: 5 µm. Insets show representative sister centromeres with four fold magnification. (**B**) JAR cells were treated with scrambled siRNA (si con, upper panel) or siRNA against survivin (si survivin, lower panel) for 36 h. Cells were then stained for tubulin, DNA, the centromere with ACA and Aurora B. The stained metaphase cells were analyzed by confocal laser scanning microscopy. Scale bar: 5 µm. Insets show representative sister centromeres with four fold magnification. (**C**) Evaluation of the distance of the sister centromeres in BeWo cells. Cells were treated as in (A) and the length of paired ACA staining was evaluated (n=52 pairs per condition) with LAS AF software. The results are presented as mean ± SD and statistically analyzed between siRNA control and siRNA survivin group. ***p < 0.001. (**D**) Evaluation of the distance between sister centromeres in JAR cells. Cells were treated as in (B) and the length of paired ACA staining was evaluated (n=50 pairs per condition) with LAS AF software. The results based on three independent experiments are presented as mean ± SEM and statistically analyzed between siRNA control and siRNA survivin group. *p < 0.05.

Using ACA (anti-centromere antibody) staining, the distance of sister centromeres, a marker of Aurora B activity in metaphase, was evaluated in trophoblastic cells. Compared to BeWo cells treated with control siRNA, the distance of sister centromeres was shorter in BeWo cells treated with siRNA against survivin (Figure 8A and C). The experiment was also performed in JAR cells and similar results were observed (Figure 8B and D). The data imply that depletion of survivin results in mis-localization and reduced activity of Aurora B in metaphase trophoblastic cells.

## Discussion

A precisely regulated proliferation, differentiation, apoptosis and invasion of trophoblasts is of essential importance for placental development during gestation. Deregulation of these processes induces disorders in pregnancy, such as preeclampsia. As a member of the CPC, an inhibitor of apoptosis, a modulator of stress and a regulator of invasion, survivin may play multiple pivotal roles in various types of placental cells throughout different stages of gestation.

In this study we show that the survivin gene is reduced in preeclamptic placentas compared to normal controls (Figure 1), which is consistent with previous results [21]. The transcription of survivin is controlled by specific sequences in its promoter: it increases during G1 and reaches its peak in G2/M [43]. Numerous factors/pathways are involved in survivin up-regulation, such as the nuclear factor-κB (NF-κB)/phosphatidylinositol 3-kinase (PI3K)/Akt pathway [44], insulin-like growth factor-1 (IGF-1)/mTOR signaling [3], members of the Ras oncogene family [45], signal transducer and activator of transcription 3 (STAT3) [46] and the anti-apoptotic factor Wnt-2 [47]. On the other hand, survivin is one of the genes repressed at the transcriptional level by p53 [48,49]. In preeclamptic placentas, NF-κB, IGF-1 and STAT3 are up-regulated [50–52], which cannot explain the decrease in the survivin gene. Intriguingly, p53 is increased in preeclampsia [20]. Whether the deregulated p53 and other tumor suppressors account for the reduction of the survivin gene in preeclamptic placentas remains to be elucidated. It will be also interesting to study if altered epigenetic modifications or deregulated microRNAs are responsible for the reduced survivin gene in preeclamptic placentas.

Unexpectedly, the protein level of survivin is not correlated to its gene level in preeclamptic placenta (Figure 2). We could not observe a clear difference in the protein level of survivin between the preeclampsia group and the control group by applying Western blot analysis and immunohistochemical staining as well (Figure 2A-F). The protein level of survivin in preeclampsia has been controversially reported, and our data are in line with one study [20] yet in contradiction with another [21]. As trophoblasts in preeclamptic placenta constantly meet stress, we assumed that the discrepancy between decreased gene and unchanged protein level of survivin could be explained by protein stabilization upon stress. Indeed, the protein survivin is stabilized upon stress in trophoblast cell lines as well as in primary trophoblast cells (Figure 3). It has been reported that stabilization of survivin could result from altered ubiquitin-proteasome pathways regulating survivin degradation [53,54], changed activation of proteins responsible for the turnover of survivin, such as Hsp90 [55], or alternatively from the post-translational modifications of survivin, such as phosphorylation [56]. Indeed, increased Hsp90 and phosphorylated serine 20 in survivin were observed in trophoblastic cells after H_2_O_2_ treatment (Figure 3A and G). Moreover, phosphorylation of the serine 20 is linked to activated PKA in trophoblastic cells upon stress (Figure 3D and H), which is in line with the observation that PKA activity is associated with increased cell survival [57,58]. Based on our data, we suggest that activated PKA and increased Hsp90 could account for stabilized survivin in trophoblasts in preeclampsia via phosphorylation and the proteasome degradation pathway, respectively. Moreover, apoptosis induction is reversely correlated with the levels of survivin (Figure 3E and F, Figure 4C and D, Figure 5A and D and Figure S2A) indicating that survivin is responsible for the survival in trophoblastic cells facing stress. It is plausible to propose that, in order to survive the stress, trophoblasts in preeclamptic placenta will turn on all the rescue mechanisms to stabilize/activate cytoprotection proteins, such as survivin. It is also conceivable to suggest that the harmful consequence will appear if the rescue mechanisms are exhausted or not able to work, possibly in the case of severe preeclampsia.

Survivin is found in villous cytotrophoblasts and syncytiotrophoblasts (Figure 2C), supporting the previous data [17,20,21]. However, while we observe the survivin signal predominantly in the nucleus (Figure 2C), the others detected the staining mostly in the cytoplasm and only rarely in the nucleus of placental cells [20,21]. To corroborate our data from immunohistochemical staining, we performed Western blot analysis using nuclear and cytosol extracts, which clearly demonstrates that the main pool of survivin in placenta cells is localized in the nucleus (Figure 2G). This discrepancy could possibly be attributed to lower sensitivity and specificity of survivin antibodies used previously by other groups. Survivin is a nuclear cytoplasmic shuttling protein [59] and these subcellular pools coincide with different survivin functions [1]. Since the nuclear pool of survivin mediates survivin’s function in mitosis [7,8], we suggest that survivin accumulated in the nucleus of cytotrophoblasts and of syncytial sprout cells is properly responsible for survival and proliferation. Moreover, it has been reported that the nuclear localization of survivin correlates with its accelerated proteosomal degradation [53] and acts as an apoptotic executor and thus commits a cell to apoptosis [60]. It is intriguing to ask whether the survivin nuclear localization observed in differentiated syncytiotrophoblasts (Figure 2C) could also promote the turnover of survivin and facilitate the fusion process. Further investigations are required to study the individual function of survivin in each cell type of the placenta.

As survivin is a member of the CPC, we have investigated its effect on mitotic progression and proliferation in trophoblasts. Indeed, survivin is critical for the viability of three trophoblast cell lines (Figure 4A, 5E and Figure S2D), regardless of malignancy or benignity. Depletion/suppression of survivin arrests trophoblastic cells in G2/M (Figure 4B, Figure 5B and C, and Figure S2B and C), followed by apoptosis (Figure 4C and D, Figure 5A and D, and Figure S2A). Furthermore, suppression of survivin in trophoblastic cells induces strong defects in chromosome congression (Figure 6), in centrosome integrity (Figure 6), in proper localization and activity of Aurora B in metaphase (Figure 7 and Figure 8). Collectively, the data demonstrate that survivin, like in tumor cells, is of essential importance for proper mitotic progression of trophoblastic cells in the placenta.

## Conclusion

Taken together, we show that the survivin gene is reduced but the protein level of survivin is hardly changed in preeclampsia placentas, in comparison with control placentas. This discrepancy between the gene level and protein amount can be explained by stabilization of the survivin protein under stress. Survivin promotes the survival of trophoblastic cells challenged by stress. It plays pivotal roles in mitotic progression in trophoblastic cells of the placenta. Interfering with survivin induces severe mitotic defects followed by apoptosis. More investigations are required to explore the function of survivin in individual cell types of the placenta. It is tempting to propose that survivin plays multifaceted roles in the placenta development in terms of proliferation, cell cycle control, apoptosis, angiogenesis and invasion. Its alteration is possibly involved in the pathogenesis of the placenta.

## Supporting Information

Figure S1 **Characterization of isolated primary trophoblasts from term placentas.** (**A**) Indirect immunofluorescence staining with antibodies against DNA, cytokeratin-7 and cytokeratin-18 (upper panel) or against DNA, vimentin and cytokeratin-18 (lower panel). Representatives are presented. Scale bar: 20 µm. (**B**) Evaluation of positive stained cells.
(TIF)

Figure S2 **Bewo cells are affected by survivin suppressant YM155.** (**A**) Western blot analysis. BeWo cells were treated with increasing concentrations of YM155 for 48 h and cellular extracts were prepared for Western blot analysis. β-actin served as loading control. Untreated (con) and DMSO treated cellular extracts were taken as controls. (**B**) Cell cycle profiles. Cells were treated as in (A) and cell cycle analysis was performed. DMSO treated cells were taken as controls. (**C**) Quantification of the sub-phases of the cell cycle. (**D**) Cell viability assay. BeWo cells were non-treated (con), treated with DMSO or with increasing concentrations of YM155 for 48 h and cell viability was analyzed. Bar: ± SD.
(TIF)
