# Supplementary figures and images for: Function of Survivin in Trophoblastic Cells of the Placenta
Source: PLoS One. 2013 Sep 19;8(9):e73337. doi: 10.1371/journal.pone.0073337 (PMC3778024; doi:10.1371/journal.pone.0073337)

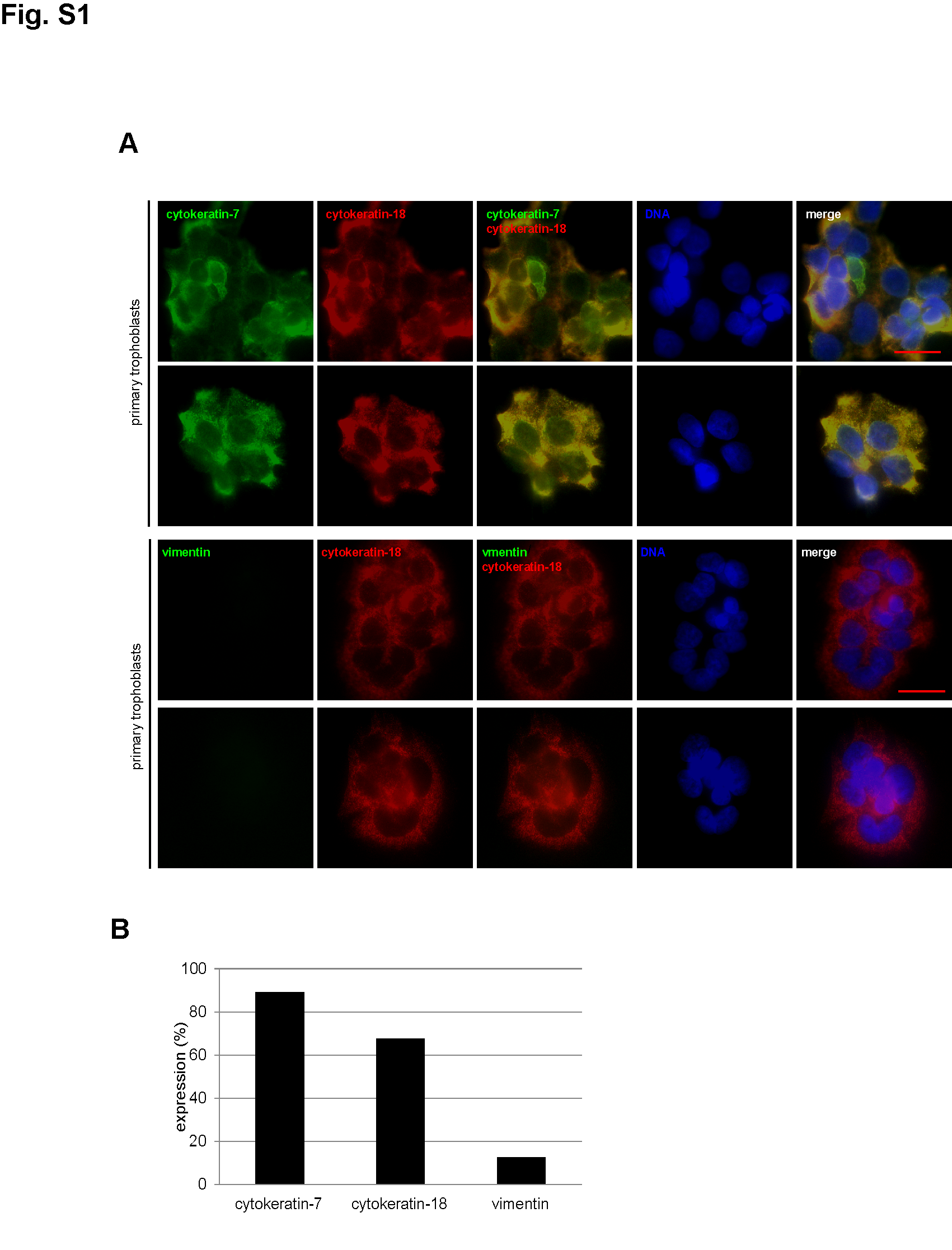

Supplement: Figure S1 — Characterization of isolated primary trophoblasts from term placentas. (A) Indirect immunofluorescence staining with antibodies against DNA, cytokeratin-7 and cytokeratin-18 (upper panel) or against DNA, vimentin and cytokeratin-18 (lower panel). Representatives are presented. Scale bar: 20 µm. (B) Evaluation of positive stained cells. (TIF) [file pone.0073337.s001.tif]

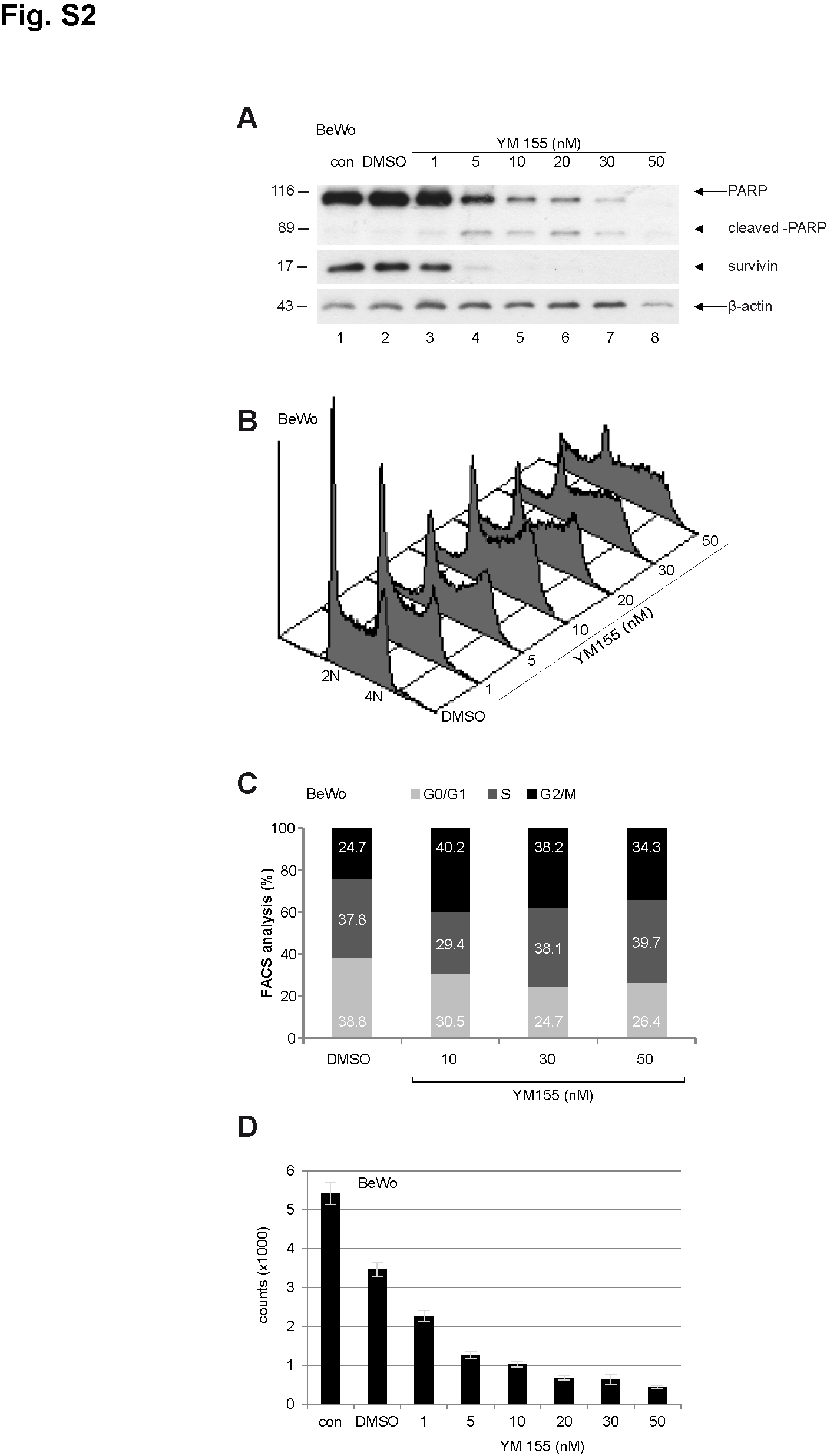

Supplement: Figure S2 — Bewo cells are affected by survivin suppressant YM155. (A) Western blot analysis. BeWo cells were treated with increasing concentrations of YM155 for 48 h and cellular extracts were prepared for Western blot analysis. β-actin served as loading control. Untreated (con) and DMSO treated cellular extracts were taken as controls. (B) Cell cycle profiles. Cells were treated as in (A) and cell cycle analysis was performed. DMSO treated cells were taken as controls. (C) Quantification of the sub-phases of the cell cycle. (D) Cell viability assay. BeWo cells were non-treated (con), treated with DMSO or with increasing concentrations of YM155 for 48 h and cell viability was analyzed. Bar: ± SD. (TIF) [file pone.0073337.s002.tif]
